# Influence of shape-memory stent grafts on local aortic compliance

**DOI:** 10.1007/s10237-021-01514-9

**Published:** 2021-09-19

**Authors:** J. Concannon, KM Moerman, N. Hynes, S. Sultan, JP McGarry

**Affiliations:** 1 Biomedical Engineering, College of Engineering and Informatics, National University of Ireland, Galway, Ireland; 2 Western Vascular Institute, National University of Ireland Galway, Galway, Ireland

**Keywords:** Aortic compliance, Collagen, Stent-graft, Finite element

## Abstract

The effect of repair techniques on the biomechanics of the aorta is poorly understood, resulting in significant levels of postoperative complications for patients worldwide. This study presents a computational analysis of the influence of Nitinol-based devices on the biomechanical performance of a healthy patient-specific human aorta. Simulations reveal that Nitinol stent-grafts stretch the artery wall so that collagen is stretched to a straightened high-stiffness configuration. The high-compliance regime (HCR) associated with low diastolic lumen pressure is eliminated, and the artery operates in a low-compliance regime (LCR) throughout the entire cardiac cycle. The slope of the lumen pressure–area curve for the LCR post-implantation is almost identical to that of the native vessel during systole. This negligible change from the native LCR slope occurs because the stent-graft increases its diameter from the crimped configuration during deployment so that it reaches a low-stiffness unloading plateau. The effective radial stiffness of the implant along this unloading plateau is negligible compared to the stiffness of the artery wall. Provided the Nitinol device unloads sufficiently during deployment to the unloading plateau, the degree of oversizing has a negligible effect on the pressure–area response of the vessel, as each device exerts approximately the same radial force, the slope of which is negligible compared to the LCR slope of the native artery. We show that 10% oversizing based on the observed diastolic diameter in the mid descending thoracic aorta results in a complete loss of contact between the device and the wall during systole, which could lead to an endoleak and stent migration. 20% oversizing reaches the Dacron enforced area limit (DEAL) during the pulse pressure and results in an effective zero-compliance in the later portion of systole.

## Introduction

Aortic disease is responsible for 47,000 deaths annually in the USA and a mortality rate following rupture of over 90% (Zimmerman et al. 2016; Kent 2014). In cases of surgical intervention, the 30-day postoperative mortality rate can be as high as 53% for endovascular aortic repair (EVAR) (Hinchliffe et al. 2006) and 48% for open surgical repair (OSR) (Greenberg et al. 2008). Several adverse events and postoperative complications associated with compliance mismatch have been reported in the literature including thrombosis (Abbott et al. 1987), false aneurysm formation (Mehigan et al. 1985) and cardiovascular complications that result in the death of the patient (Nauta et al. 2017). These figures and complications are particularly concerning given that although the majority of patients who undergo aortic repair are elderly, a large number of young patients (< 30 years of age) are also treated for various aortic pathologies including aneurysm and dissection (Yoneyama et al. 2019; Marder, Aird, and White 2012; Sörelius 2016; Hountis et al. 2009; Tiryakioglu et al. 2009; Abbaszadeh, Eslami, and Nikparvar 2019). Furthermore, a substantial cohort exists who undergo stenting of an *otherwise healthy aorta* to treat aortic transection, aorto-enteric fistula, and penetrating aortic ulcer (Nation and Wang 2015; Lonn et al. 2010; Kotsis et al. 2019) in young in addition to degenerative atherosclerotic disease in old patients (Donas et al. 2011).

In the case of EVAR, a stent-graft (Nitinol frame and a polytetrafluoroethylene or woven polyester graft) is deployed intravascularly with the aim of (1) reducing of stress in an aorta/aneurysm wall, or (2) preventing further propagation of an aortic dissection and removing the false lumen. Several aortic stent-graft designs are available commercially, and in this study, we show that our implicit effective stent membrane (ESM) approach to modelling open cell and braided stents provides similar results to lengthy explicit simulations that are required to deal with the complex contact/buckling issues that arise during the crimp and deployment steps of the procedure. In the case of OSR, the diseased section of the aorta is removed and replaced with a synthetic vessel. The graft, generally fabricated from polyethylene terephthalate or Dacron, comprises of tightly woven or knitted fibres with high tensile strength (Sarkar et al. 2006) and is sutured (proximally and distally) to the remaining native aortic tissue.

It has not been established whether EVAR or OSR is the superior treatment option for patients with aortic aneurysm or dissection. A meta-analysis of 42 non-randomised studies evaluating EVAR versus OSR for descending thoracic aortic disease reported no significant difference between intervention groups in relation to stroke, reintervention and mortality beyond 12 months (Cheng et al. 2010). A multi-centre prospective study comparing EVAR to OSR for a cohort of 341 thoracoabdominal aortic aneurysm patients found no statistical difference between groups in relation to 30-day mortality or paraplegia (Tshomba et al. 2017). Most recently, a study by Salata et al. (2019) reports no statistically significant difference between outcomes after EVAR and OSR to repair abdominal aortic aneurysms in the long-term mortality during more than 13 years of follow-up. What is clear, however, is that both treatment options dramatically alter the natural biomechanics of the aorta.

Experimental flat-plate compression and radial crimping of commercially available grafts have been reported by De Bock et al. (2013). Biaxial tension tests of graft materials demonstrate that stent-grafts are up to 25 times stiffer than the healthy aorta (Tremblay et al. 2009). Pressure inflation tests also reveal that Dacron is significantly stiffer circumferentially than native aortic tissue (Ferrari et al. 2019), while Bustos and Celentano (2016) report a circumferential strain of less than 3% for Dacron grafts subjected to a pressure of 240 mmHg. Singh and Wang (2015) suggest that current commercially available stent-grafts act as a rigid non-distensible conduit and fail to replicate the natural deformation of the aorta.

This study presents a computational analysis of the influence of Nitinol-based devices on the biomechanical performance of a ‘non-aneurysmal’ patient-specific human aorta (e.g. following aortic transection, aorto-enteric fistula, penetrating aortic ulcer). An MRI-based subject-specific aortic model (Concannon et al. 2020) and an aortic constitutive law based on elastin pre-strain, SMC contractility and collagen strain stiffening (Concannon and McGarry 2021) are used as a platform to assess the alterations in the lumen pressure–area relationship due to clinical intervention. The study uncovers a number of fundamentally important, and previously unreported, insights that should be of critical concern for device design and clinical practice. Novel computational analyses reveal the effects of device oversizing on biomechanical performance of young and old aortae. We also uncover the importance of patient age/aortic compliance, and deployment location on the clinical selection of an appropriate graft size.

## Model development

### Mechanical behaviour of Aortic Stent-Grafts

**Fig. 1:**
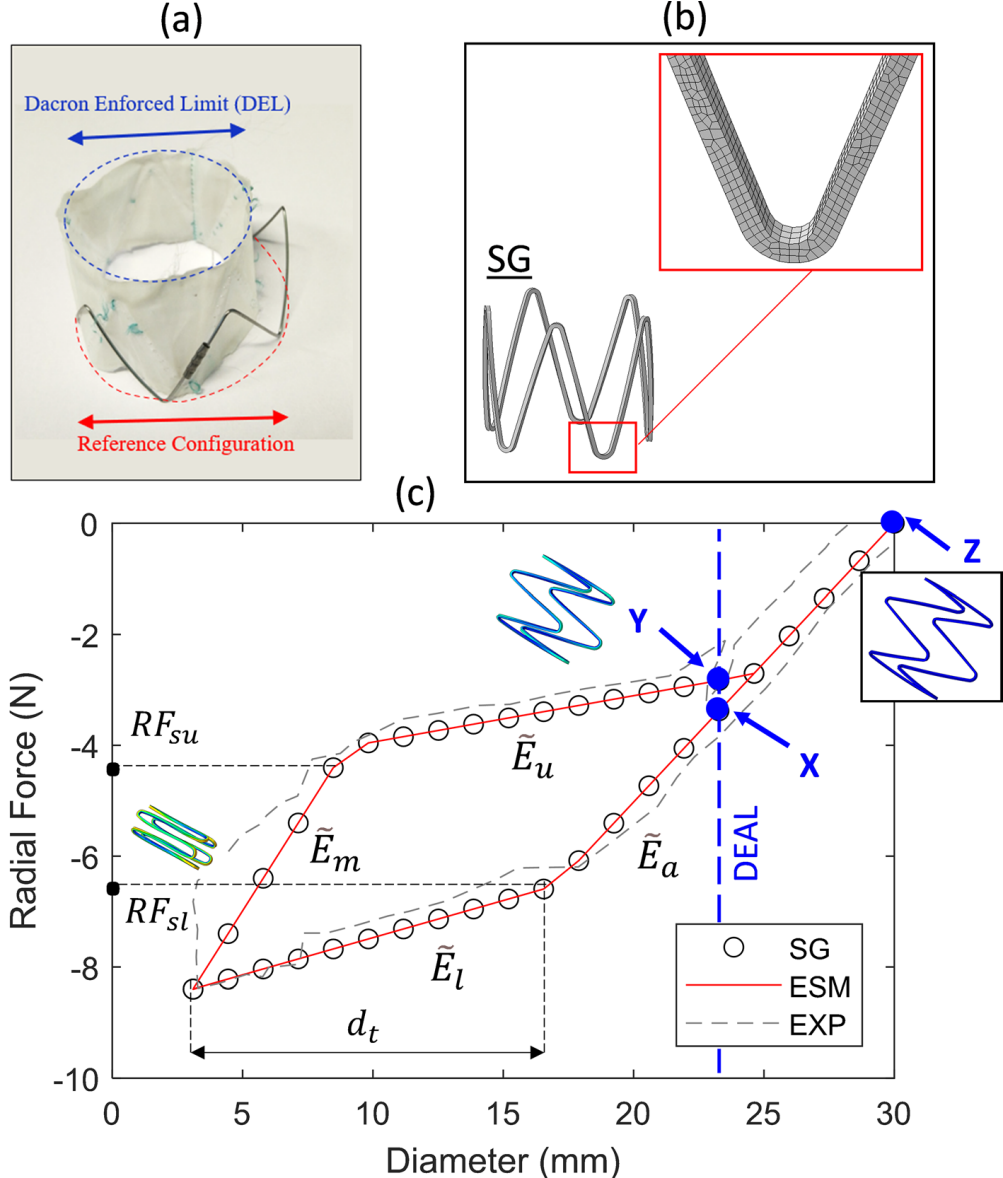
**a** By removing the stitches that attach the NiTi stent rings to the graft material, we can observe that the diameter of the stent expands by a further 24%, to the true stress-free reference configuration. In vivo, the DEAL prevents the implant from returning to this reference configuration. **b** A finite element model of the stent graft (SG) is created and crimp and deploy steps are simulated. **c** The RF-D relationship of the SG model is recorded, and the effective stent membrane (ESM) model is created and subjected to the same displacement boundary conditions. Calibration of the parameter values of the ESM constitutive model results in an identical RF-D response throughout the crimp and deployment steps to that of the full stent-graft model. The reference configuration (point Z) is the configuration of the stent once it has been removed from the Dacron graft. Both the SG and ESM are then crimped to the internal diameter of the delivery sheath. During the deployment step, the implant expands until the Dacron enforced area limit (DEAL) is reached, after which the implant has essentially a zero-compliance due to the stiffness of the Dacron (point Y). The experimental test results of (Zhou et al. 2019) are superimposed for comparison (EXP)

*Identifying the reference stress-free device configuration:* Removal of the stitching which ties the Nitinol frame to the graft material reveals that the stent is not in a stress-free configuration when attached to a fully expanded Dacron graft. Rather, removal of the graft results in a 24% increase in the effective diameter of the stent-ring (Fig. 1a). To accurately simulate such devices, it is important to base all calculations on this stress-free reference state. *The device diameter indicated in the product catalogues is not the stress-free configuration; rather, it is the diameter that is enforced by the stiff graft material to which the stent is stitched.* Throughout this study, we refer to this configuration as the Dacron enforced area limit (DEAL) or the Dacron enforced diameter limit (DEDL). The fully expanded Nitinol stent geometry is taken to be the stress-free reference configuration.

We begin by performing a simulation of the crimp and deploy of a Nitinol stent-graft (SG) ring, using the inbuilt Abaqus/Standard Superelastic shape-memory material definition (Auricchio and Taylor 1997). The material parameters and values are presented in Table 1, and the stent-graft geometry is based on the commercially available Valiant Captivia system (Medtronic Vascular, Santa Rosa, CA, USA) with an unloaded stress-free (reference configuration) diameter of 30 mm. The finite element model and close-up of the mesh are shown in Fig. 1b. The full stent graft radial force (RF) versus device diameter (D) history during the crimp and release/deployment cycle is presented in Fig. 1c. The experimental results of Zhou et al. (2019) are also superimposed for comparison, which together highlight several key features of the crimp/deploy process. During crimping, the device initially reduces its diameter according to the austenitic device radial stiffness (DRS). At a given diameter (in this case ~ 17 mm), the stent begins transformation from austenite to martensite at localised regions. Further crimping results in a lower apparent DRS until the maximum crimp diameter is reached. Subsequent release/uncrimping/deployment initially results in unloading with a DRS which differs from the initial loading DRS due to (1) the presence of martensite, and (2) geometric deformation of the device. At a diameter of ~ 9 mm, transformation from martensite to austenite begins and again the DRS is considerably lower until such a diameter that the phase transformation is complete, after which the curve follows the initial austenitic DRS back to the stress-free reference configuration.

**Table 1:** Nitinol parameters and values applied to SG model (inbuilt Abaqus Superelastic Shape-Memory material formulation (Auricchio and Taylor 1997))

Parameter	Description	Value
${\mathbf{E}}_{{\mathbf{a}}}$	Austenite Young’s Modulus	53,001 (MPa)
${\varvec{\nu}}_{{\varvec{a}}}$	Austenite Poisson’s Ratio	0.3
${\mathbf{E}}_{{\varvec{m}}}$	Martensite Young’s Modulus	21,500 (MPa)
${\varvec{\nu}}_{{\varvec{m}}}$	Martensite Poisson’s Ratio	0.3
${\varvec{\varepsilon}}_{{\varvec{t}}}$	Transformation Strain	0.038
${\varvec{\sigma}}_{{\varvec{L}}}^{{\varvec{S}}}$	Start of Transformation Loading	434 (MPa)
${\varvec{\sigma}}_{{\varvec{L}}}^{{\varvec{E}}}$	End of Transformation Loading	500 (MPa)
${\varvec{\sigma}}_{{\varvec{U}}}^{{\varvec{S}}}$	Start of Transformation Unloading	210.3 (MPa)
${\varvec{\sigma}}_{{\varvec{U}}}^{{\varvec{E}}}$	End of Transformation Unloading	138.7 (MPa)
${\varvec{\sigma}}_{{{\varvec{CL}}}}^{{\varvec{S}}}$	Start of transformation stress in compression	434.0 (MPa)
${\varvec{\varepsilon}}_{{\varvec{V}}}^{{\varvec{L}}}$	Volumetric Transformation Strain	0.038

The description above, however, related to Fig. 1 does not consider the effect of the DEAL. The Dacron graft (once stitched) prevents the SG from ever returning to the stress-free reference configuration. During the crimp phase, the graft does not play a role mechanically as it is in a buckled/wrinkled state. During deployment, this is also the case, until the DEAL is reached (after which the DRS of the system significantly increases to give an effective zero-compliance). Figure 1c highlights the operational range of the device which starts at point X (the constrained SG diameter due to the Dacron), follows the load/unload curve to Y (the point at which the deal is reached during unloading), but never back to Z (the stress-free reference configuration). As a consequence, it is important to note that at a diameter below the DEAL, the reaction force experienced by the artery is due to the SG; however, at a diameter above the DEAL, the reaction force of the stent is only transferred to the Dacron and not to the artery. In Fig. 1c, the effective DRS history is shown, where $\tilde{E}_{a}$ represents the austenitic phase, $\tilde{E}_{l}$ the transformation loading phase, $\tilde{E}_{m}$ the martensitic phase, and $\tilde{E}_{u}$ the transformation unloading phase. The diameter-change during transformation loading, in addition to the radial force at start of transformation loading ($RF_{sl}$) and start of transformation unloading ($RF_{su}$), is also highlighted.

*Homogenised effective stent membrane (ESM) modelling approach for efficient analysis of graft performance:* Owing to the considerable computational cost of modelling complex geometric details of stent-graft geometries (e.g. braided stent designs or closed cell stent designs), we next present a homogenised stent-graft model that can be calibrated such that the radial force (RF) versus diameter (D) relationship is identical to that of a full stent (SG) model described above. A phenomenological constitutive law is developed and implemented in a user material subroutine (UMAT) so that the following features of a full device are replicated: the initial high-stiffness regime during loading; the low stiffness loading plateau; the initial unloading regime; the low stiffness unloading plateau. For the remainder of this paper, we refer to this homogenised device modelling approach as the effective stent membrane (ESM). The graft material only contributes mechanically once the DEAL is reached; during the crimp and deployment (up until the DEAL is reached), the graft is in a wrinkled/buckled state between the stent struts. Due to the extremely high stiffness of the graft material (2.9 GPa (Santos et al. 2012)), negligible axial deformation is incurred over the crimp/deploy step and hence, we prescribe an effective device Poison’s ratio of zero to the ESM. A cylindrical orientation defines the circumferential, axial and normal direction of each M3D4 membrane element. As part of the current study, we demonstrate that this computationally efficient ESM approach can be used as a preliminary design tool in the development/validation of stent graft performance based on the required/measured effective radial force–diameter characteristics of the device.

In addition to the commercially available stent-graft described above and shown in Fig. 1c, we also consider in subsequent analyses two other stent-graft sizes to investigate the effects of oversizing on the pressure–area relationship of the aorta. As discussed later, we model stents of sizes 22, 26, and 30 mm. Similar RF-D curves are generated for 20, 40, and 60% oversizing cases.

### Artery constitutive model and capturing *in vivo* nonlinearity

The constitutive model for the aorta employs a rule of mixtures approach where each component of the wall undergoes the same strain, but the stresses are additive, and the sum of constituent densities equals unity. The constituents of the wall explicitly modelled include collagen, elastin, SMCs, and ground-matrix. A bilinear strain energy function describes the strain stiffening behaviour of fibrillar collagen under tension

1 $$ \sigma_{{{\text{col}}}} = V_{{{\text{col}}}}^{f} * \left\{ {\begin{array}{*{20}l} 0 \hfill & {\varepsilon < 0} \hfill \\ {\left( {k_{1} \varepsilon } \right) * \left( {a_{{{\text{col}}}} \otimes a_{{{\text{col}}}} } \right)} \hfill & {0 \le \varepsilon < \varepsilon_{t} } \hfill \\ {\left( {k_{1} \varepsilon + k_{2} \left( {\varepsilon - \varepsilon_{t} } \right)} \right) * \left( {a_{{{\text{col}}}} \otimes a_{{{\text{col}}}} } \right)} \hfill & {\varepsilon_{t} \le \varepsilon } \hfill \\ \end{array} } \right. $$

where $V_{{{\text{col}}}}^{f}$ is the volume fraction of collagen in the wall, $k_{1}$ is the initial stiffness of collagen, $k_{2}$ is the secondary stiffness of collagen, $\varepsilon_{t}$ is the transition strain, $\varepsilon$ is the strain, and ${\varvec{a}}_{{{\text{col}}}}$ denotes the current direction of deformed collagen fibres with respect to the circumferential axis of the artery, where ${\varvec{a}}_{{{\text{col}}}} = {\mathbf{F}}{\varvec{a}}_{{{\text{col}}}}^{0}$ (${\mathbf{F}}$ = deformation gradient, and ${\varvec{a}}_{{{\text{col}}}}^{0}$ is the direction of undeformed collagen fibres in the reference configuration) (Concannon and McGarry 2021). Elastin fibres may also contribute to the anisotropy of the vessel with a mean directionality defined by ${{\varvec{a}}}_{ela}$. The elastin fibres also exhibit a constant pre-stretch (${\lambda }_{e}=1.6$) and stiffness and to allow contraction of the vessel such that the stress is

2 $$ {\varvec{\sigma}}_{{{\text{ela}}}} = V_{{{\text{ela}}}}^{f} *\left( {\left( {\sqrt {I_{4e} } - 1 + \lambda_{e} } \right)*k_{e} } \right)*\left( {{\varvec{a}}_{{{\text{ela}}}} \otimes {\varvec{a}}_{{{\text{ela}}}} } \right). $$

The model parameter $\lambda_{e}$ is the pre-stretch of the elastin component in the initial undeformed configuration. $I_{4e}$ is the elastin fibre anisotropic invariant, i.e. the square of stretch of an elastin fibre ($I_{4e} = \left( {{\varvec{a}}_{{{\text{ela}}}} \cdot {\text{C}}{\varvec{a}}_{{{\text{ela}}}} } \right)$, where ${\text{C}}$ is the right Cauchy–Green tensor). As described above for collagen, ${\varvec{a}}_{{{\text{ela}}}} = {\mathbf{F}}{\varvec{a}}_{{{\text{ela}}}}^{0}$. For anisotropic invariant of the type${I}_{4k}$, we use full invariant rather than the isochoric invariant. This is necessary for reasons outlined in our previous paper (Nolan et al. 2014). $V_{{{\text{ela}}}}^{f}$ is the volume fraction of elastin in the wall, and ${k}_{e}$ is the stiffness of elastin. A linear relationship between ${I}_{4e}$ and Cauchy stress is supported by the experimental evidence of (Aaron and Gosline 1981; Gundiah et al. 2009), where a linear stress–stretch relationship is observed for ${I}_{4e}$ =2 (extension of 100%) for an elastin fibre. In each of the simulations presented here, ${I}_{4e}$ is less than 1.5 indicating that that we are comfortably within the linear regime for elastin.

We also incorporate the active component of the wall as represented by ${\sigma }_{smc}$. The contractile stress generated by a single SMC (${\sigma }_{act}$) is 25 kPa (McGarry et al. 2009). SMCs also contribute to the anisotropy of the vessel through ${{\varvec{a}}}_{smc}$

3 $$ {\varvec{\sigma}}_{{{\text{smc}}}} = V_{{{\text{smc}}}}^{f} *\left( {\sigma_{{{\text{act}}}} *\left( {{\varvec{a}}_{{{\text{smc}}}} \otimes {\varvec{a}}_{{{\text{smc}}}} } \right)} \right) $$

where $V_{{{\text{smc}}}}^{f}$ is the volume fraction of SMCs in the wall, $\sigma_{{{\text{act}}}}$ is the active stress of an individual SMC and ${\varvec{a}}_{{{\text{smc}}}} = {\mathbf{F}}{\varvec{a}}_{{{\text{smc}}}}^{0}$. Finally, the stress in the isotropic ground-matrix can be defined as:

4 $$ {\varvec{\sigma}}_{{{\text{mat}}}} = V_{{{\text{mat}}}}^{f} *\left( {\frac{{\mu_{0} }}{{J^{\frac{2}{3}} }}\left( {{\overline{\mathbf{B}}} - \frac{1}{3}\overline{I}_{1} {\mathbf{I}}} \right) + \frac{2J}{{D_{1} }}\left( {J - 1} \right)} \right) $$

where ${V}_{mat}^{f}$ is the volume fraction of matrix in the wall, ${\mu }_{0}$ is the shear modulus, and ${D}_{1}$ is related to the bulk modulus of the ground-matrix. $\overline{\mathrm{B} }$ is the isochoric left Cauchy–Green tensor, ${\overline{I} }_{1}$ is the trace of the isochoric left Cauchy–Green tensor, $J$ is the determinant of the deformation gradient, and $\mathbf{I}$ is the identity matrix. Throughout this study, we assume a circumferential alignment of collagen, elastin and SMCs following extensive analysis which highlights that anisotropy and vessel layer heterogeneity have a second-order effect on the pressure–area relationship. For further information, the reader is referred to Concannon and McGarry (2021).

**Fig. 2:**
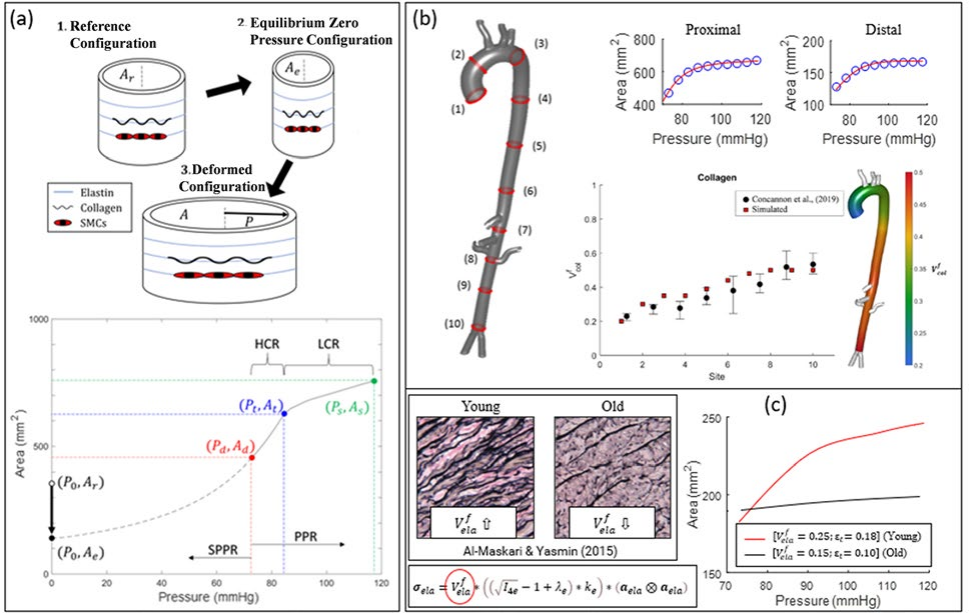
Overview of the key features of the MRI/FEA framework that is critical to the following study. **a** 1. A reference configuration is constructed with an area ${\mathbf{A}}_{\mathbf{r}}$. 2. An equilibrium zero-pressure configuration is computed, whereby the cross-sectional area of the vessel reduces to ${\mathbf{A}}_{\mathbf{e}}$, such that the internal tensile circumferential stress due to elastin pre-stretch and SMC contractility is in equilibrium with the compressive circumferential stress generated in the matrix. 3.) The lumen pressure is increased from zero up to the peak systolic value (117 mmHg). The pressure increases through a sub-physiological pressure regime (SPPR). At the diastolic pressure, ${\mathbf{P}}_{\mathbf{d}}$, (73 mmHg) the computed lumen area is denoted ${\mathbf{A}}_{\mathbf{d}}$. The pressure is then increased through the physiological pressure range (PPR) up to the peak systolic value, ${\mathbf{P}}_{\mathbf{s}}$ (117 mmHg), at which point the computed area is denoted ${\mathbf{A}}_{\mathbf{s}}$. **b** The model is shown to accurately capture the spatially varying MRI data simply by calibrating the spatially varying volume fractions of the constituents (${\mathbf{V}}_{\mathbf{c}\mathbf{o}\mathbf{l}}^{\mathbf{f}}$, ${\mathbf{V}}_{\mathbf{e}\mathbf{l}\mathbf{a}}^{\mathbf{f}}$, ${\mathbf{V}}_{\mathbf{s}\mathbf{m}\mathbf{c}}^{\mathbf{f}}$), and the transition strain of the collagen. **c** In this study, we investigate the effects of stenting on the biomechanics of the healthy (Young) aorta, in addition to an artificially aged (Old) aorta. By reducing the volume fraction of elastin by 10% and reducing the collagen transition strain by 0.15 in accordance with the experimental studies of (Hosoda et al. 1984 and Vande Geest and Vorp. 2004), we obtain the ‘Old’ aorta which exhibits a stiffer and less nonlinear response across the physiological pressure range compared to the young aorta

Figure 2 provides an overview of the key features of the MRI/FEA framework that is critical to the following study in terms of predicting *in vivo* deformation using our histologically motivated constitutive law. Firstly, Fig. 2a(1.) depicts a reference configuration constructed with an area A_r_. (2.) An equilibrium zero-pressure configuration is computed, whereby the cross-sectional area of the vessel reduces to A_e_, such that the internal tensile circumferential stress due to elastin pre-stretch and SMC contractility is in equilibrium with the compressive circumferential stress generated in the matrix. 3.) The lumen pressure is increased from zero up to the peak systolic value (117 mmHg). The pressure increases through a sub-physiological pressure regime (SPPR). At the diastolic pressure, P_d_, (73 mmHg) the computed lumen area is denoted A_d_. The pressure is then increased through the physiological pressure range (PPR) up to the peak systolic value, P_s_ (117 mmHg), at which point the computed area is denoted A_s_. As illustrated in the pressure–area curve above, based on our MRI measurements, the PPR is characterised by a high-compliance regime (HCR) up to a transition pressure, P_t_, followed by a low compliance regime (LCR) up to the peak of systole. In Figure 2b, the spatially varying pressure–area relationships along the length of the aorta can then be predicted by altering the volume fractions of the wall constituents, mainly collagen, elastin and SMCs, as well as the collagen transition strain (ε_t_). Excellent agreement is observed between the predicted volume fractions of wall constituents along the aorta and histological investigations by the same authors on cadaveric aortae (Concannon et al. 2019). In this study, we investigate the effects of stenting on the biomechanics of the healthy (Young) aorta, in addition to an artificially aged (Old) aorta. By reducing the volume fraction of elastin by 10% and reducing the collagen transition strain by 0.15 in accordance with the experimental studies of (Hosoda et al. 1984 and Vande Geest and Vorp. 2004), we obtain our ‘*Old*’ aorta (Fig. 2c) which exhibits a stiffer, almost linear response across the physiological pressure range compared to the young highly compliant and nonlinear aorta. We observe an A_s_/A_d_ ratio of 1.1 (where A_s_ and A_d_ indicate the cross-sectional area at systole and diastole), which compares favourably with the experimental study of Hallock and Benson (1937) who found an A_s_/A_d_ = 1.09 for the 71–78-year-old cohort. For comparison, the same study reports an A_s_/A_d_ = 1.39 for the 20–24-year-old cohort, compared to an A_s_/A_d_ = 1.42 in our healthy 26-year-old male volunteer.

### Comparison of ESM and Stent-Graft on Artery Compliance

**Fig. 3:**
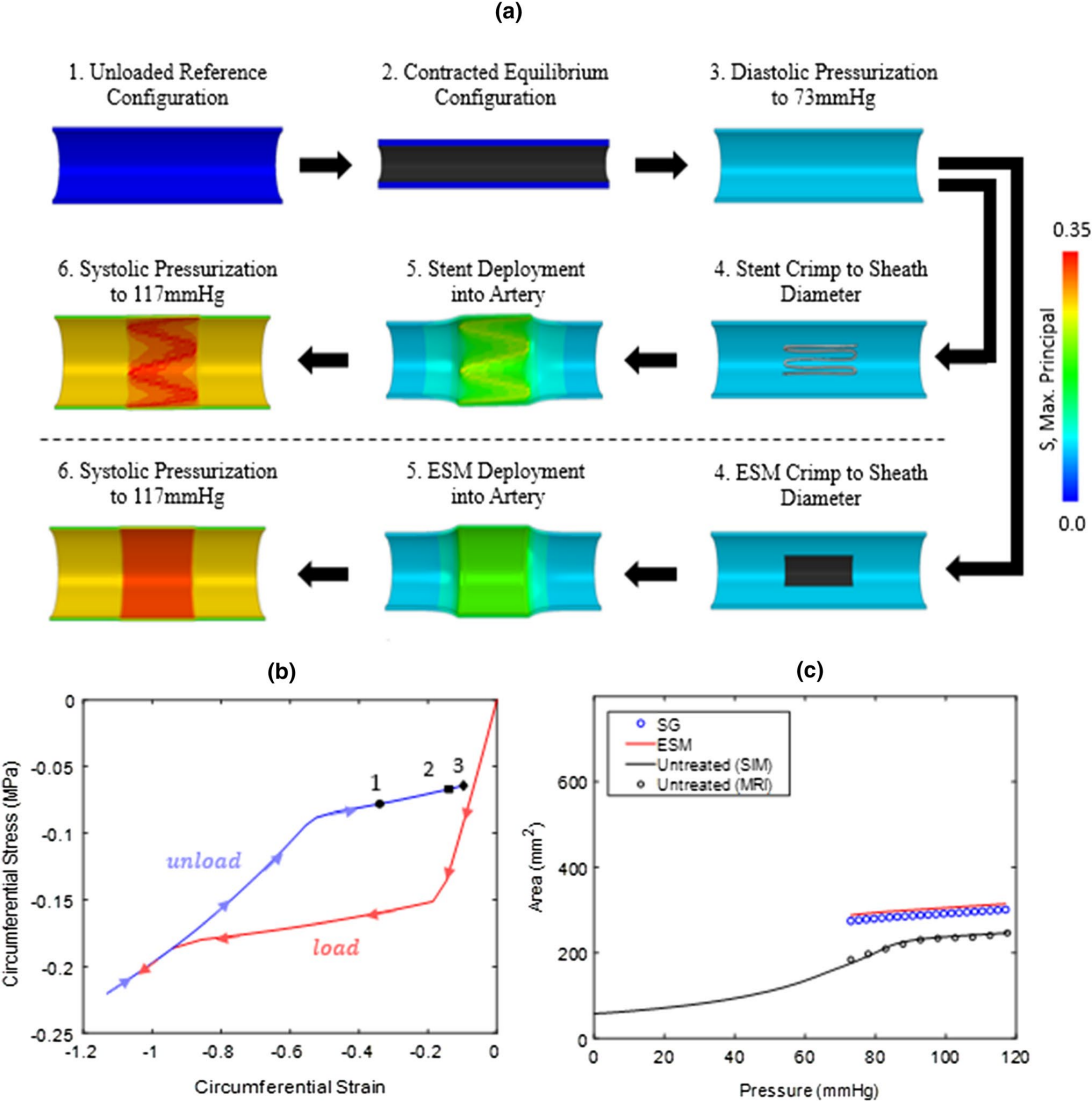
**a** Flow chart for solution procedure. (1.) The unloaded reference configuration of the artery. (2.) The contractile elements within the arterial wall result in a reduction in area and a new equilibrium configuration. (3.) Diastolic pressure of 73 mmHg is applied to the internal lumen surface. (4.) The SG/ESM is crimped to the inner sheath diameter of the delivery device. (5.) The SG/ESM is deployed into the artery, which remains under the diastolic pressure load, and a new equilibrium area is reached. (6.) The system is pressurised with a pulse pressure to bring the total applied lumen pressure to 117 mmHg. **b** Circumferential stress versus strain plot for the ESM model shows that the device come in contact with the arterial wall at (1) along the ‘unloading plateau’, which results in an increase in the diastolic equilibrium area (2). By applying the pulse pressure, the NiTi behaviour follows the high compliance stiffness of the unloading plateau, which is significantly less than the LCR stiffness of the aorta. **c** Both models (ESM and SG) predict that the lumen area is ~ 53% higher than the untreated artery at diastolic pressure, and ~ 24% higher than the untreated artery at peak systolic pressure. As the effective compliance of the implant is negligible compared to the high stiffness of the low compliance regime (LCR) of the aorta, a similar slope is observed in the pressure–area relationship between the untreated LCR and the area increase due to the pressure pulse post-implantation

**Fig. 4:**
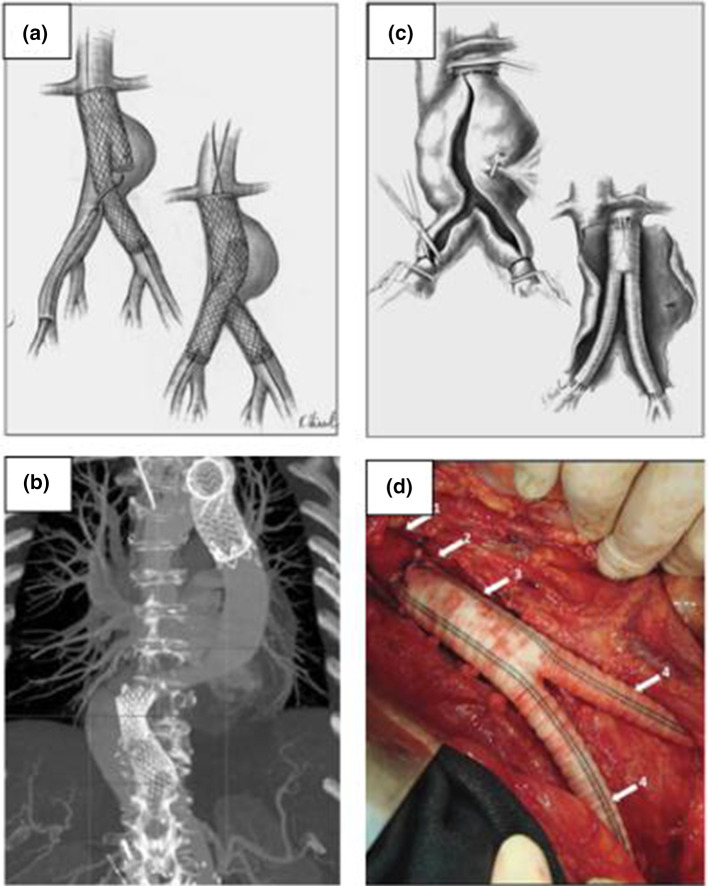
Available treatment options for aortic disease. **a**, **b** Endovascular aortic repair (EVAR); **c**, **d** open surgical repair (OSR). **a** A schematic of the EVAR procedure where a stent-graft is deployed intravascularly to the disease site (Figueroa and Zarins 2011). **b** Postoperative CT scan showing proximal aortic and distal aortic stents (Sultan and Hynes 2014). **c** A schematic of the OSR procedure where the healthy aorta proximal and distal to the diseased site are clamped, the diseased portion is cut open and a synthetic graft is sutured into the native vessels proximally and distally (Figueroa and Zarins 2011). **d** Perioperative image of OSR of infrarenal aorta with Dacron graft (Baila et al. 2016)

The solution procedure for the FE simulations is as follows. As described in (Concannon and McGarry 2021), the equilibrium zero-pressure configuration due to elastin pre-stretch and smooth muscle cell (SMC) contractility is computed. The internal lumen surface is then loaded to a diastolic pressure of 73 mmHg. The SG and ESM are then crimped to the internal diameter of the delivery sheath. Deployment of the SG and ESM is simulated by simply removing the crimp boundary condition. As the implant expands, it comes into contact with the vessel wall, leading to a further increase in the vessel diameter until an equilibrium deployed configuration is achieved, whereby the outwards radial force of the device and the lumen pressure is in equilibrium with the inwards force due to the circumferential tensile stress in the stretched contractile artery wall. Finally, the lumen pressure is increased to the systolic value of 117 mmHg. As shown in Fig. 3, results for the SG and ESM models are compared in terms of the maximum principal stress (S) distribution in the artery wall, and in terms of the pressure–area relationship post-deployment. The ESM simulation exhibits an approximately uniform distribution of stress in the peri-implant artery wall. The SG simulation exhibits a similar stress state, but with higher stress concentrations in the regions where the struts directly contact the artery. The pressure–area curves computed for the SG and ESM are extremely similar. Both models predict that the lumen area is ~ 53% higher than the untreated artery at diastolic pressure, and ~ 24% higher than the untreated artery at peak systolic pressure. This indicates that the ESM model provides sufficient accuracy for assessment of a device induced compliance alteration. The ESM approach does not, however, provide a detailed description of stress concentrations in the vessel wall due to complex geometries of stent struts. It should be noted that in the open-cell stent design considered here, a low percentage of the overall device area consists of stent struts, generating high concentrations of stress in the regions of strut-vessel contact. In contrast, braided stent designs, or closed-cell designs, such as those shown in Fig. 4a, b, are commonly used for aortic repair. The high area fraction of strut coverage for such designs suggests that the ESM modelling strategy will provide a very accurate description of the stress distribution in the vessel wall, in addition to accurately predicting of the effective vessel compliance.

Both the SG and the ESM models predict that the characteristic bilinear shape of the pressure–area relationship of the untreated aorta is eliminated following deployment of the device. Instead, a linear pressure–area curve is obtained following device deployment, the slope of which is similar to the slope of the low compliance regime (LCR) of the untreated artery in the systole pressure range. Analyses reveal that the primary effect of deployment of the Nitinol stent is to expand the artery so that collagen fibres are in the high-stiffness regime throughout the entire cardiac cycle. The fact that the device does not significantly alter the pressure–area slope from the native LCR slope can be explained by considering the circumferential stress–strain path computed during the crimp-deployment history of the device, as shown in Fig. 3b. As the device is deployed (from its crimped state), it initially reduces its strain at a high modulus (the value of which is primarily governed by the martensite modulus of the NiTi stent). However, when the device reaches its equilibrium configuration at the diastolic pressure, it deforms along the “unloading plateau”, which has an extremely low effective circumferential tangent stiffness of ~ 0.07 MPa. The device unloading profile remains on this plateau up to the point of maximum systolic pressure. Given that the effective tangent modulus of the artery in the LCR is ~ 1 MPa, the insertion of the device does not significantly change the effective compliance from the native vessel LCR. Rather, the only significant effect of the device is elimination of the native vessel high-compliance regime (HCR) during diastole. Once again, we emphasize that this occurs simply because the device stretches the collagen in the vessel wall into a high-stiffness regime for the entire cardiac cycle. The stiffness of the device becomes significant only if the DEAL is reached during physiological loading, in which case the device becomes extremely stiff compared to the native vessel, and the effective compliance of the treated section becomes zero for part of the cardiac cycle. In Sect. 2.4, we investigate the critical issue of device over-sizing and the selection of an appropriate DEAL.

**Fig. 5:**
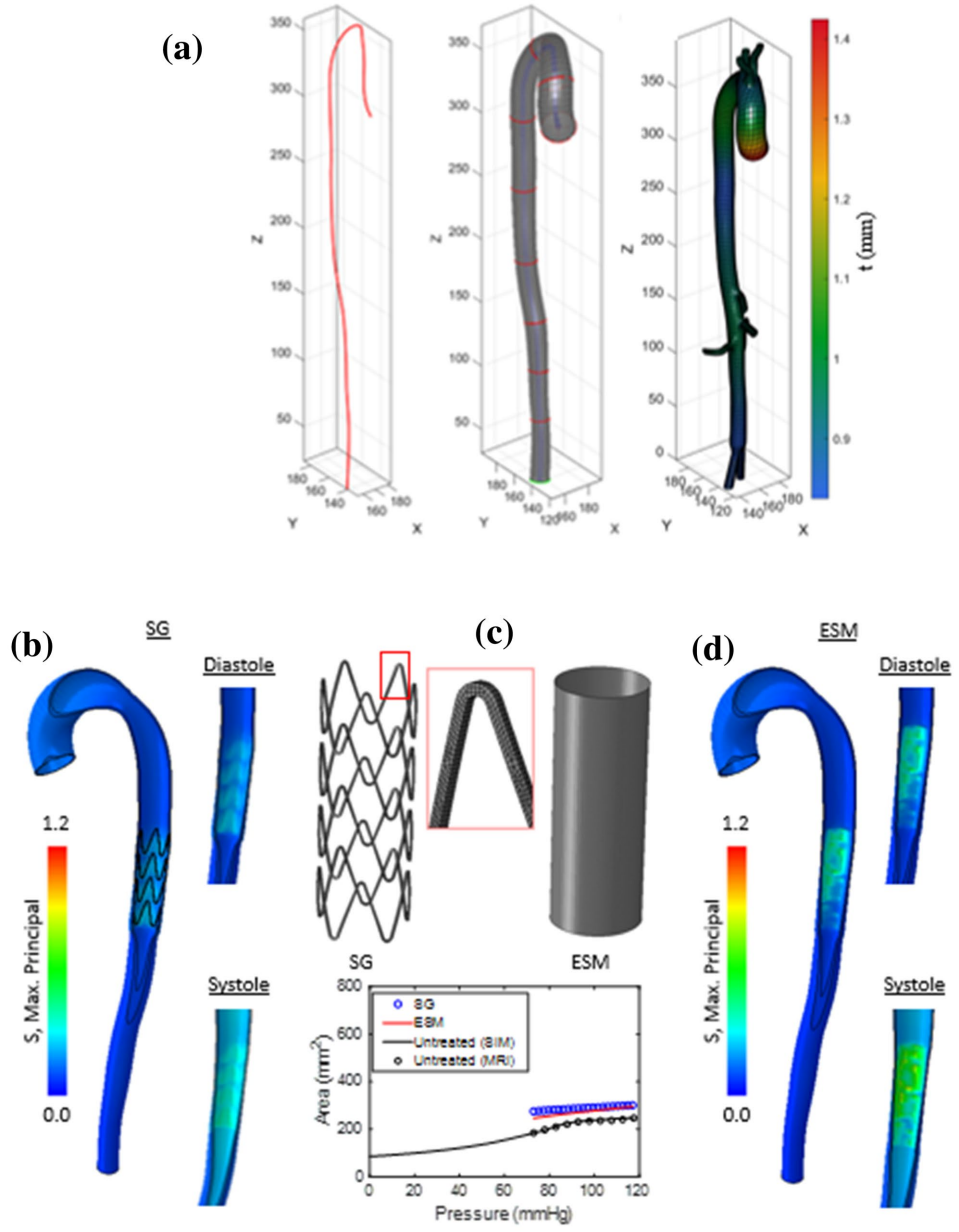
**a** Finite element mesh generation directly from MRI dataset. Heterogeneous wall thickness (t) is implemented based on histology data from Concannon et al., (2019). **b**-**d** Comparison of SG and ESM models in a subject-specific aorta. **b** Deployment of the SG results in stress concentrations in the regions where the struts directly contact the artery wall. **c** Both the SG and ESM models show a similar prediction of the implant-induced compliance alteration on the pressure–area relationship. **d** The ESM simulation exhibits more uniform distribution of stress in the peri-implant artery wall; however, the level of stress is similar to that of the SG model

In Fig. 5, we present a comparison of the SG and ESM models deployed in a subject-specific FE model of the aorta. This patient-specific mesh was generated using MATLAB (R2017b, MathWorks Inc., Natick, MA) and GIBBON (Moerman 2018), by sweeping a mesh containing the aortic lumen boundary points at 10 planes along the aortic centreline (Fig. 5a), both of which were determined from a 4D Flow MRI scan of a young healthy volunteer (Concannon et al. 2020). Regional wall thickness was interpolated to each node along the aortic mesh based on the histological study by Concannon et al. (2019) resulting in a highly structured model comprised of ~ 42,000 C3D8 elements. (This mesh size was found to give a converged solution in terms of computed spatially varying nonlinear compliance with three elements through the thickness of the aortic wall.) The *in vivo* nonlinear aortic compliance was captured using the physical based constitutive law outlined in Sect. 2.2 (Concannon and McGarry 2021) with the following material parameter values: ${k}_{1}$ = 0.2 MPa, ${k}_{2}$ = 1.0 MPa, ${k}_{e}$ = 0.035 MPa, ${\lambda }_{e}$ = 1.6, ${V}_{col}^{f}$ = 0.20–0.50, ${V}_{ela}^{f}$ = 0.18–0.35, ${V}_{smc}^{f}$ = 0.20–0.27, ${\varepsilon }_{t}$ = 0.15–0.38, ${\mu }_{0}$ = 0.10–0.80, ${\sigma }_{act}$ = 25 kPa. We assume that the fundamental material properties that describe collagen ($k_{1} ,k_{2}$), elastin ($k_{e} ,\lambda_{e}$) and SMCs ($\sigma_{{{\text{act}}}}$) do not change as a function of location along the aorta. Rather we assume that the volume fractions of collagen ($V_{{{\text{col}}}}^{f}$), elastin ($V_{{{\text{ela}}}}^{f}$), SMCs ($V_{{{\text{smc}}}}^{f}$) and matrix ($V_{{{\text{mat}}}}^{f}$) may vary spatially based on previous experimental evidence. The model is primarily calibrated by altering $V_{{{\text{col}}}}^{f}$, $V_{{{\text{ela}}}}^{f}$,$V_{{{\text{smc}}}}^{f}$, and the transition strain ($\varepsilon_{t}$), ensuring a unique parameter set.

In the patient-specific case, a longer stent graft consisting of four stent rings, is considered. Figure 5b, d shows the maximum principal stress (S) plotted along the aorta for both the SG and ESM simulations, respectively. The ESM model provides a reasonable approximation of the stress state in the vessel wall, compared to the SG model. The volume-averaged mean values of maximum principal stress, ($\sigma_{{{\text{maxP}}}}^{{{\text{avg}}}}$), and strain, ($\varepsilon_{{{\text{maxP}}}}^{{{\text{avg}}}}$), in the vessel wall in the stented region are found to be similar for models of the stent graft ($\sigma_{{{\text{maxP}}}}^{{{\text{avg}}}}$ = 0.239 MPa; $\varepsilon_{{{\text{maxP}}}}^{{{\text{avg}}}}$ = 0.257) and ESM ($\sigma_{{{\text{maxP}}}}^{{{\text{avg}}}}$ = 0.246 MPa; $\varepsilon_{{{\text{maxP}}}}^{{{\text{avg}}}}$ = 0.231). However, as shown in Fig. 5b, the ESM model does not capture localised concentrations, as observed for SG simulations. Once again, as shown in Fig. 3c, both devices provide similar predictions of the altered pressure–area relationship following implantation. As was the case in the idealised cylinder models described above, the device is found to stretch the subject-specific artery wall in the circumferential direction so that the collagen is stretched beyond the transition strain (${\upvarepsilon }_{\mathrm{t}}$) at all times during the cardiac cycle. This results in the elimination of the HCR of the pressure–area curve. Once again, the effective compliance of the stented section is extremely similar to that of the native vessel during systole because the device follows the relatively low unloading plateau during the increase of the lumen pressure from diastole to systole. The comparisons presented in Figs. 3 and 5 suggest that the ESM modelling approach provides a reasonable approximation to the predicted behaviour of a full stent model—even for the low density open-cell geometry considered here.

### Effect of implant oversizing and positioning on compliance

We next use the validated ESM modelling approach to investigate the influence of device over-sizing, device positioning, and native vessel age on the post-implantation changes in the aortic pressure–area relationship.

**Fig. 6:**
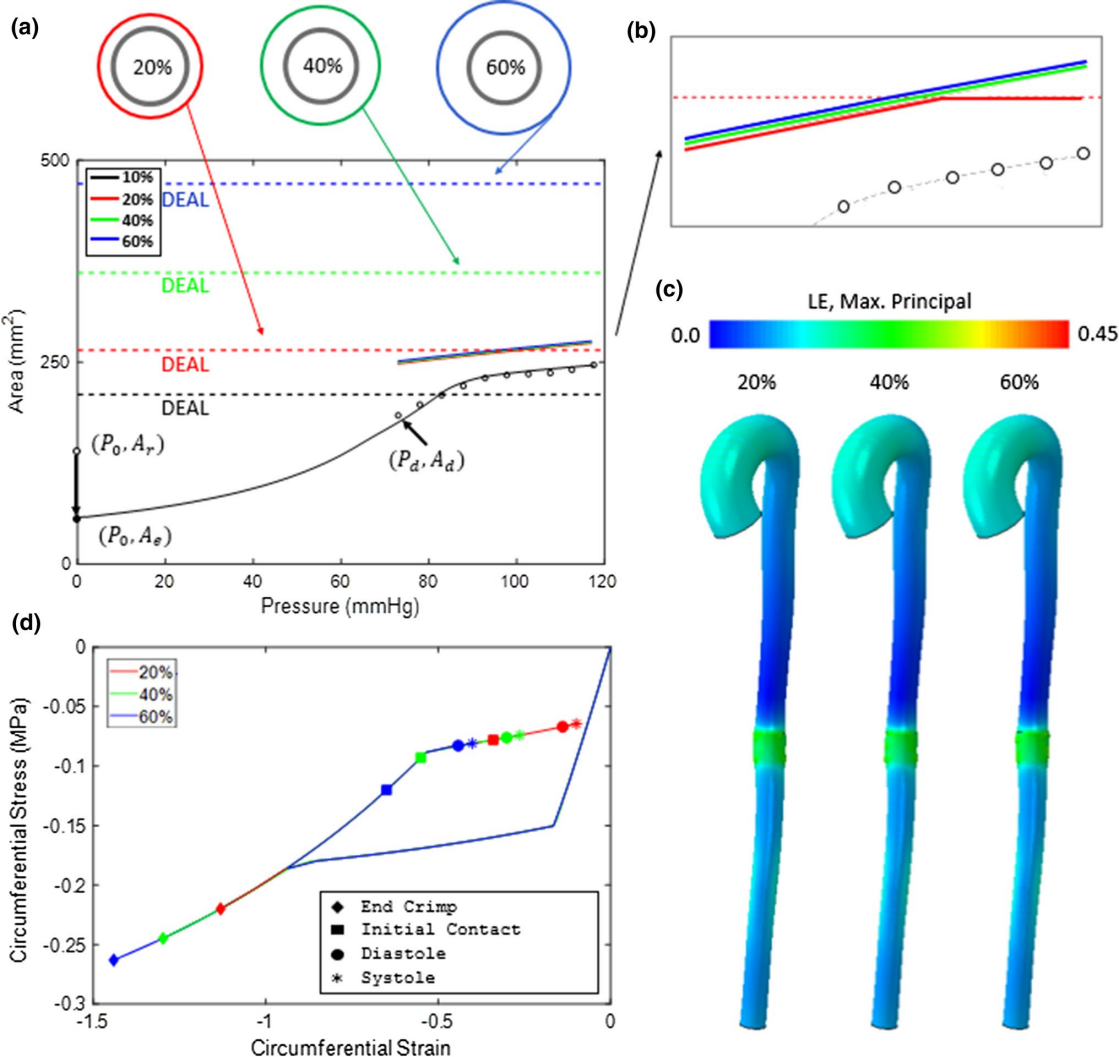
**a** The effects of ESM oversizing on the pressure–area relationship in Plane 6 of the human aorta. In each case, the 20% (red), 40% (green) and 60%(blue) oversizing results in any area gain due to pulse pressure to follow the secondary stiffness slope of the aorta. **b** In the case of 20% oversizing, the DEAL is indicated by the dashed red line, below which the pressure–area response follows the secondary stiffness of the aorta. Once the DEAL is reached, no further area gain is incurred for any further increase in pressure (as indicated by the change in slope of solid red line at ~ 93 mmHg). **c** Finite element contour plot of max. Principal logarithmic strain (LE), highlighting similar strain levels at the deployment site for each degree of oversizing. This can be explained by considering the device effective circumferential stress–strain history during crimping and deployment **d**. In each case, the diastolic equilibrium configuration lies on the unloading plateau where the stiffness of the Nitinol is negligible compared to that of the secondary stiffness of the native aorta. With the application of the pulse pressure, each degree of oversizing remains on the unloading plateau and therefore exerts approximately the same radial force on the arterial wall

*Influence of Oversizing—Model development:* The effect of EVAR device oversizing on the pressure–area relationship at Plane 6 (mid descending thoracic aorta) is investigated using the subject-specific aortic model. Clinical guidelines recommend 10–20% oversizing for aortic stent-grafts (Sher and Tadros 2017; van Prehn et al. 2009). Here, we consider three device designs in which the Dacron enforced diameter limit (DEDL) exceeds the diastolic vessel diameter by 20, 40 and 60%, as illustrated in Fig. 6. Based on commercially available designs, in all cases the diameter of the stent stress-free configuration is taken to be 24% greater than the Dacron graft (i.e. the DEDL). Table 3 shows the diameter of the stress-free stent configuration, the DEDL and the crimped diameter for the three device designs under consideration. The crimp diameter is based on the use of a standard commercially available 22F delivery system (Ramanan et al. 2015). In all cases, the crimped device is deployed at section 6 of the subject-specific aortic model, where the vessel area is 184 mm^2^ at the diastolic pressure of 73 mmHg and 247 mm^2^ at the peak systolic pressure of 117 mmHg. For each of the three stent designs, FE simulations of crimping and deployment are performed to determine the RF-D relationship. Corresponding ESM models are then calibrated to replicate the stent RF-D relationship (Table 2) and deployed in the subject-specific artery.

**Table 2:** Parameters for ESM model deployed into patient-specific aorta

Parameter	Description	Value
${\tilde{E }}_{a}$	Effective device radial stiffness (austenite phase)	0.60 (N/mm)
${\tilde{E }}_{m}$	Effective device radial stiffness (martensite phase)	0.50 (N/mm)
${\tilde{E }}_{l}$	Effective device radial stiffness (transformation loading phase)	0.10 (N/mm)
${\tilde{E }}_{u}$	Effective device radial stiffness (transformation unloading phase)	0.07 (N/mm)
$R{F}_{sl}$	Device radial force at start of transformation loading phase	10.5 (N)
$R{F}_{su}$	Device radial force at start of transformation unloading phase	6.20 (N)

*Influence of Oversizing—Results:* Figure 6a shows the post-implantation pressure–area curves at section 6 of the subject-specific aorta for the 20, 40 and 60% oversized devices. The DEAL for each case is also indicated on the figure. Nearly identical pressure–area curves are computed for all three designs, the only difference being that the 20% design reaches the DEAL at a pressure of 93 mmHg, after which the compliance is effectively zero. Post-implantation areas at the start of diastole and end of systole are listed in Table 3 for all three devices. The finding that the biomechanical outcome is essentially independent of the degree of device oversizing can be understood by considering the device effective circumferential stress–strain history during crimping and deployment, as shown in Fig. 6d. While the 60% oversized device reaches the highest compressive strain during crimping, as expected, all three devices are compressed to a strain that extends beyond the loading plateau. Therefore, all three devices follow the same unloading curve during deployment in the vessel. Furthermore, at the new diastolic equilibrium state, all three devices have reached the unloading plateau. Therefore, while the device diastolic strain is sensitive to the degree of oversizing (i.e. position along unloading curve), the circumferential stress of each device is similar, so therefore the effective radial force and the diastolic vessel diameter are similar for all three cases. Finally, all three devices remain on the unloading plateau as the pressure is increased to the end-systolic value. Therefore, the effective stiffness of the device is extremely low compared to the vessel, and the slope of the pressure–area curve throughout the cardiac cycle is essentially equal to the native vessel LCR slope for all three device designs.

**Table 3:** Geometrical parameters for the 20%, 40% and 60% oversized implants

	Reference Configuration Diameter (mm)	DEDL (mm)	Crimp diameter (mm)	Post-Operative diastolic Area (mm^2^)	Post-Operative systolic area (mm^2^)
20%	22.0	18.4	7.1	248	273
40%	26.0	21.4	7.1	250	275
60%	30.0	24.5	7.1	252	276

The influence of the DEAL should be emphasized. As described above, the 20% oversized device reaches the DEAL at a lumen pressure of 93 mmHg, so the device becomes effectively, infinitely stiff and the vessel area remains constant as the pressure is increased to the peak-systolic value (i.e. the compliance is near zero in this regime). *We recommend that the device should be sufficiently oversized so that the DEAL is not reached during physiological loading and a zero-compliance regime does not occur*, as is the case for the 40% and 60% oversized devices. Excessive oversizing (> 60%) may have two negative drawbacks: (1) the device may not reach the unloading plateau when deployed, in which case it will exhibit a higher tangent stiffness and the effective vessel compliance will be lower than the native LCR; (2) the device may not fit in a standard delivery catheter, or it may fracture during crimping. However, insufficient oversizing may be catastrophic. As an example, we illustrate in Fig. 6 that the DEAL associated with a 10% oversized device is lower than the lumen area during systole. This presents a significant risk of endoleak and device migration.

**Fig. 7:**
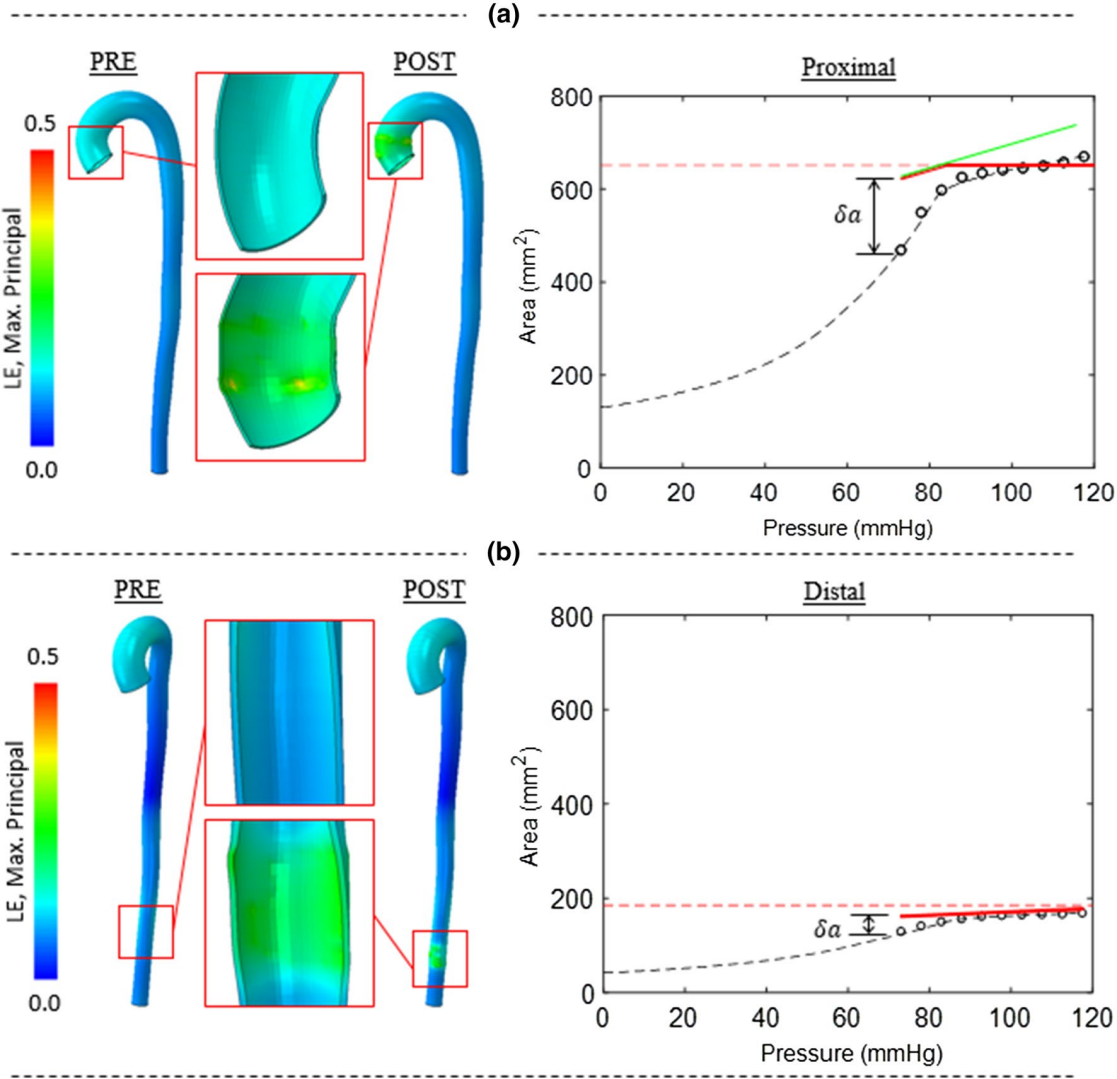
20% oversized stent-graft in the **a** proximal aorta and **b** distal aorta. Pre-operative pressure–area relationship is indicated by open circles and dashed line (fitted). Dotted line indicates the DIAL when the stent-graft is fully expanded, and no further area increase can be incurred for a given increase in pressure

Figure 7 shows the effect of implant deployment on the pressure–area relationship in the human aorta proximally (7(a)) versus distally (7(b)). At the proximal section, the effective compliance of the vessel post-implantation is similar to the LCR of the native vessel. It should be noted that the native vessel at this proximal section is the most compliant in the aorta. Therefore, the elimination of the extremely high-compliance diastolic regime following device implantation results in a significant reduction in lumen area change during a cardiac cycle (ΔA = 202 mm^2^ for the native vessel, versus ΔA = 28 mm^2^ post-implantation, representing an 86% reduction). Simulations also reveal the critical importance of device oversizing in highly compliant proximal sections: as shown in Fig. 7, the DEAL for a 20% oversized device is lower than the end-systolic area of the native vessel, indicating a high risk of endoleak and migration. A 40% oversizing is required for this compliant proximal section.

Figure 7b shows the simulated behaviour for the distal section of the aorta. The compliance of the native vessel at this section is the lowest in the aorta. In this case, a 20% oversized device does not reach the DEAL and the stented vessel exhibits a compliance similar in value to the native LCR for the entire duration of the cardiac cycle. In this case, the reduction in area change during the cardiac cycle is not as significant as the effect proximally (ΔA = 38 mm^2^ for the native vessel, versus ΔA = 13 mm^2^ post-implantation, representing an 65% reduction).

**Fig. 8:**
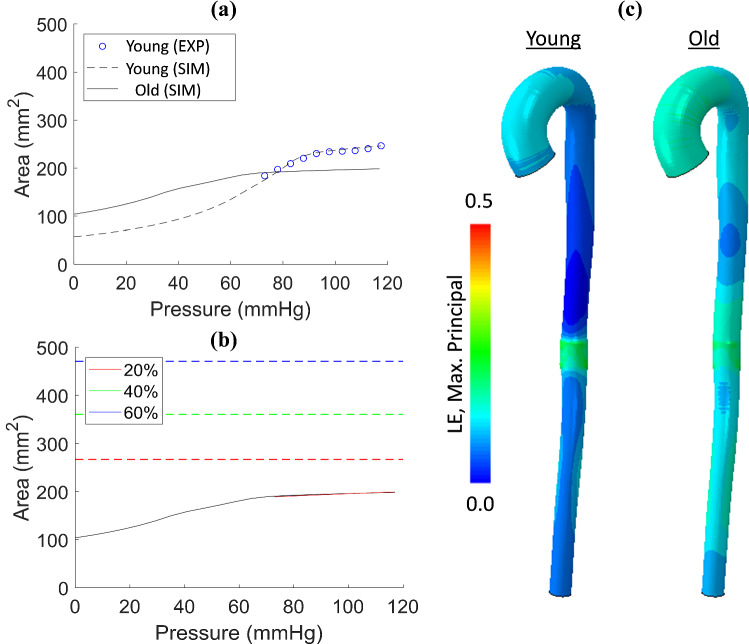
**a** Healthy (Young) pressure–area relationship for Plane 6 as indicated by blue open circles, and FE fit (dashed black line) compared to Old (solid black line). The Old aortic properties were achieved by reducing both the elastin volume fraction within the wall and the transition strain. **b** The effect of implant oversizing on the pressure–area relationship in an Old aorta. 20, 40 and 60% oversizing are indicated by the solid red, green and blue lines, respectively. The DIAL for each percentage oversizing is indicated by the dashed red, green and blue lines, respectively. **c** Finite element mesh of subject-specific aorta comparing healthy (Young) and pathologically stiffened (Old) properties in terms of max. Principal logarithmic strain (LE)

The relationship between vessel compliance and device design is further elucidated in Fig. 8, in which we simulate age-related arteriosclerotic stiffening of the aorta by reducing the volume fraction of elastin by 10% throughout the entire vessel (see Eq. 4 in our constitutive law in (Concannon and McGarry 2021)), based on the histological study of Hosoda et al. (1984). We also reduce the transition strain at which collagen stiffening occurs (see Eq. 3 in (Concannon and McGarry 2021)), based on reported biomechanical testing of aged and young aortic tissue (Vande Geest and Vorp 2004). The stiffened aortic pressure–area relationship for Plane 6 is shown in Fig. 8a. Compared to a healthy ‘Young’ tissue, the ‘Old’ aorta does not exhibit a bilinear pressure–area curve under the physiological range of lumen pressures due to the reduction of the collagen transition strain, i.e. an LCR-type regime occurs throughout the entire cardiac cycle. The reduction of elastin also increases the zero-pressure reference area, further adding to the linear shape of the pressure–area curve and the overall reduction in vessel compliance. For such a stiffened artery, the insertion of a stent-graft does not significantly alter the pressure–area curve of the vessel. The stiff vessel is already in the high-stiffness collagen regime, so the area change and compliance change effected by device insertion are negligible, again because the deployed devices operate on the unloading plateau of the RF-D curve. In this case, a 20% over-sizing is sufficient as the DEAL is not reached during the cardiac cycle.

## Discussion and concluding remarks

This study presents a computational analysis of the influence of Nitinol-based devices on the biomechanical performance of the aorta. Specifically, the influence of device implantation on the pressure–area curve of the vessel is analysed. The study uncovers a number of fundamentally important, and previously unreported, insights that should be of critical concern for device design and clinical practice.

1. The Nitinol stent-graft device expands the artery wall into a new equilibrium configuration which exceeds the transition strain of the vessel, meaning that the artery is now in the low-compliance regime (LCR) where the high stiffness of straightened collagen fibres governs the mechanical behaviour of the wall, and the high-compliance regime (HCR) at diastolic pressure is eliminated.
2. The stent-graft unloads from the crimped configuration during deployment and operates along the unloading plateau between diastole and systole. The direct effective stiffness of the implant is negligible compared to the high stiffness of the artery wall in the LCR. As a result, the pressure–area relationship post-stenting between diastole and systole follows that of the local LCR slope of the aorta.
3. Provided the Nitinol device increases its diameter sufficiently during deployment so that it reaches the unloading plateau, the degree of oversizing has a negligible effect on the pressure–area response of the vessel. Each oversized device considered in this study is found to exert approximately the same radial force because all devices reach the unloading plateau. Additionally, the radial compliance (the slope of the radial force-diameter curve) on the unloading plateau is negligible compared to the LCR slope of the native artery.
4. We show that 10% oversizing based on the observed diastolic diameter in the mid descending thoracic aorta results in a complete loss of contact between the device and the wall during systole, which could lead to an endoleak and stent migration. 20% oversizing reaches the Dacron enforced area limit (DEAL) during the pulse pressure and results in an effective zero-compliance in latter portion of systole.
5. Our study suggests that oversizing is more important proximally, while stiffer distal vessels do not require as much oversizing. The implanted section follows the local LCR slope for the entire cardiac cycle, and as a result, the effective compliance change in diastole is more pronounced in proximal sections where the difference between the HCR and LCR of the native vessel is greatest.
6. Device deployment in a pathologically stiffened (‘Old’) artery (in which the collagen transition strain and elastin content are both decreased) does not strongly alter the lumen pressure–area curve. Furthermore, oversizing of the device has a negligible effect on the lumen pressure–area curve.

Following EVAR, the Nitinol implant expands the artery wall into a new equilibrium configuration whereby the outwards radial force of the device and the lumen pressure is in equilibrium with the inwards force due to the circumferential tensile stress in the stretched contractile artery wall. In this configuration, where the circumferential strain of the vessel wall exceeds the collagen transition strain, the artery operates in the low-compliance regime (LCR) throughout the entire cardiac cycle. The high stiffness of the stretched/straightened collagen fibres governs the mechanical behaviour of the wall in both diastole and systole. Furthermore, as the implant unloads from the crimped configuration during the deployment step, it operates along the unloading plateau between diastole and systole, and therefore, the direct effective stiffness of the implant is negligible compared to the high stiffness of the artery wall in the LCR. As a result, the pressure–area relationship between diastole and systole follows that of the local LCR slope of the aorta. The deployment of the device merely expands the vessel diameter so that collagen in the vessel wall is in the high-stiffness regime throughout the cardiac cycle and the compliance of the vessel itself is decreased during diastole. Several studies, both *in vivo* and *in vitro,* report that the deployment of self-expanding stent-grafts reduces aortic wall compliance (Morris et al. 2016; Nauta et al. 2017; Vernhet et al. 2001; Back et al. 1994). However, to the author’s knowledge, this study is the first to report the fundamental mechanism by which this occurs, and the first to report that device deployment alters the compliance only at low pressures during diastole.

Clinical guidelines recommend 10–20% oversizing for aortic stent-grafts based on the vessel diameter (Sher and Tadros 2017; van Prehn et al. 2009). However, we show in (Concannon et al. 2020) that in the native aorta exhibits an area change between 15 and 65% from the start of systole to the end of systole. Other studies report similar levels of *in vivo* area change during a cardiac cycle (Sonesson et al. 1994; Sugitani et al. 2012; Ferruzzi and Humphrey 2013; Kim et al. 2019) providing substantial evidence that clinicians cannot be certain that a 10% oversized device will maintain contact with the aortic wall throughout the entire cardiac cycle. Further uncertainty is introduced through the routine use of non-cardiac-gated imaging modalities for preoperative planning and device sizing, in that it is not known whether the pre-intervention imaging of the vessel shows the configuration at diastole or at peak systole, or at an unknown intermediate lumen pressure. The size of the stent-graft relative to the vessel diameter presents a significant design challenge. As outlined above, undersizing may lead to the DEAL, beyond which essentially negligible area increase is incurred for a given increase in pressure due to the extremely high stiffness of the Dacron graft. Additionally, undersizing can also lead to an inadequate seal resulting in endoleak and/or migration. Oversizing overcomes these obstacles; however, it also has drawbacks in terms of graft folding/buckling. One possible solution may exist in replacing the stiff Dacron material with a more compliant material that strains and unstrains with the deformation of the system; however, extensive experimental/computational analysis on such a next generation device would be necessary.

Provided the Nitinol device increases its diameter sufficiently during deployment to reach the unloading plateau, the degree of oversizing has a negligible effect on the pressure–area response of the vessel, as approximately the same radial force is generated by each device. The radial compliance (the slope of the radial force–diameter curve) on the unloading plateau is negligible compared to the LCR slope of the native artery; therefore, the device itself does not significantly contribute to the overall compliance of the section. Rather, the device merely stretches the collagen in the vessel wall into a straightened stiff configuration, thus eliminating the HCR associated with wavy collagen in diastole. Our study provides an explanation for the observations of an experimental study by Nauta et al*.* (2017), where porcine aortae were connected to a mock circulatory loop and the radial strain was recorded following the deployment of three oversized stent-grafts (0–10%, 10–20%, and 20–30%). Experiments revealed that the device oversizing does not significantly affect the vessel radial strain.

Insufficient device oversizing has catastrophic consequences including device migration and endoleak, resulting in pulsation, dilation and rupture of the aneurysmal wall (Chuter 2002). We show that 10% oversizing based on the observed diastolic diameter in the mid-descending thoracic aorta results in a complete loss of contact between the device and the wall during systole. Our study suggests that significant oversizing is critical in proximal regions where the compliance of the native vessel is highest. Our findings are supported by the results of a clinical trial conducted by Donas et al. (2019) who found that patients who received stent-grafts that were oversized by 14–20% had higher incidences of Type 1a (proximal) endoleaks requiring reintervention than patients that were oversized by 22–30%. Furthermore, the Eurostar data report that the rate of endoleak decreases as the degree of oversizing increases from 0–20%, after which it is reported to plateau (Chuter 2002). Our study suggests that a higher plateau of oversizing (> 20%) is appropriate for vessel sections that exhibit higher compliance, e.g. cases where devices must be deployed in proximal sections, or cases where devices must be deployed in younger subjects.

To the best of the author’s knowledge, this is the first study to report the Dacron enforced area limit (DEAL), and its implications on vessel biomechanics. The role of the graft is to: (1) de-pressurize the aneurysm wall leading to a reduced risk of rupture; (2) hold the stent rings together; and (3) act as a conduit for blood and pressure transfer. When the device is being deployed, the graft itself does not contribute mechanically until the DEAL is reached due to it being in a crimped/buckled configuration which provides no outwards radial force. Once the DEAL is reached, however, the high stiffness of the Dacron material (~ 3 GPa) results in an effective zero-compliance of the device, whereby negligible area increase will occur for a given pressure increase. Guan et al. (2016) report no significant difference in the diameter change of Dacron stent-grafts between the mid-ring region (where the struts are sutured to the Dacron) and inter-ring region (space between two consecutive axial stent rings) when subjected to an internal pressure, indicating the Dacron material governs the mechanical response of the device once the DEAL is reached. Overall, the authors report a 0.02-mm radial displacement following the application of internal lumen pressures up to 150 mmHg. It is worth noting that there is no DEAL on braided stents, and the proposed ESM methodology should be extremely suitable for modelling such devices where the Nitinol is uniformly distributed throughout the device with a higher area fraction than the open-cell design chosen here. Owing to the extensive computational costs in modelling braided stents, the ESM method may provide an alternative approach in clinical-based finite element modelling of devices where speed of simulation is critical.

The effects of longitudinal pre-stress were investigated previously (Concannon and McGarry 2021). As can be observed in the abovementioned study, the *in vivo* nonlinear compliance curves can be captured with a similar degree of accuracy for the homogeneous and trilayered wall models (with an axial stress component). As the behaviour of the wall follows the local LCR slope due to straightened collagen (${k}_{2}$) (which is the same between both models), it can be appreciated that longitudinal pre-stress will not significantly alter the results. The focus of this study was to investigate the effect of stent-graft deployment on the radial deformation of the aorta, which lead to crucial insights in terms of device–vessel interactions. As described previously, following deployment, the implanted section follows the behaviour of the local low compliance regime (LCR) during the entire cardiac cycle. As a result, the effective change in diastole following device deployment is more pronounced in proximal sections than in distal sections distally. This is the case simply because the difference between the HCR and LCR is found to be highest in proximal sections. This finding is supported by the experimental study of Nauta et al*. *(2017). Furthermore, clinical studies report incidences of cardiac events in 34–45% of patients that undergo stenting of the thoracic aorta (Conrad et al. 2017; Beach et al. 2017; Bischoff et al. 2016; Martín et al. 2008). Such incidences are considerably lower in abdominally stented patients 6–12% (Atti et al. 2018; Blankensteijn et al. 1998; Barakat et al. 2015; Dakour Aridi et al. 2018). The findings of our computational investigation may provide an explanation for such clinical outcomes by demonstrating that proximal device deployment results in a more pronounced change in effective compliance in diastole that is the case for distal device deployment.

Our results show that device deployment in pathologically stiffened arteriosclerotic (‘Old’) arteries results in a less pronounced effect on the pressure–area curve than the healthy control case. Furthermore, oversizing has a negligible effect on the pressure–area curve as the baseline stiffness in the old aorta is higher resulting in an almost identical diastolic equilibrium configuration following deployment. This result provides an explanation for the observation by Nauta et al*.* (2017) that the radial strain is similar both pre- and post-stenting in a naturally stiffer abdominal aorta. It should be noted that older aortae are generally wider and more tortuous, which was not accounted for in this model. We simulate the arteriosclerotic aorta by altering the material compliance based on previously published histological evidence. Future studies should focus on material characterization of older patients preoperatively, followed by stent-graft deployment simulations to investigate this effect in more detail.

Several experimental studies, in addition to clinical trials, show that stent-grafts increase the pulse wave velocity (PWV) of the aorta (de Beaufort et al. 2017; Kadoglou et al. 2012, 2014; Liam Morris et al. 2013; Van Noort et al. 2018), which is an independent risk factor for cardiac failure (Ben-Shlomo et al. 2014). Importantly, the Windkessel effect is diminished following stenting (Nauta et al. 2017; Belz 1995), and increased PWV has been directly linked to cardiac death via a complex cascade of increased pulse pressure (Benetos et al. 1997), increased left-ventricular afterload and decreased coronary flow (Spadaccio et al. 2016; Zacharoulis et al. 2007), and ultimately left ventricular hypertrophy (Kim et al. 1995; Morita et al. 2002).

We have demonstrated that the key biomechanics of the problem can be uncovered by coarse-graining the device geometry. It should be noted, however, that even if all anatomical complexities of the vessel and geometrical complexities of the stent are eliminated by simulating a simplified ESM device in a perfectly axisymmetric cylindrical vessel, an analytical solution cannot be identified due to the complex nonlinear material behaviour (collagen strain stiffening, SMC contractility, elastin pre-stretch, Nitinol phase transformation) and finite deformations. In our patient-specific finite element model, we also incorporate heterogeneity in mechanical properties, in addition to contact mechanics at the device–vessel interface.

Thoracic endovascular aortic repair (TEVAR) and abdominal endovascular aortic repair (EVAR) are associated with reduced early perioperative morbidity and mortality compared to open surgical repair. However, this early gain is diminished at long-term follow-up, primarily due to an upsurge in cardiovascular complications, mainly related to the graft material (Sultan et al. 2019; Sultan, Acharya, and Hynes 2020; Hynes et al. 2020; Sultan, Concannon et al. 2020). The failure of synthetic grafts to simulate the natural biomechanics of the aorta results in a cascade of haemodynamic and biological shifts that disturb cardiovascular homeostasis (Sultan Barrett et al. 2020a, b, c; Sultan et al. 2015; Hynes et al. 2020). Currently available aortic stent-grafts have been implicated in the development of acute systolic hypertension, elevated pulse pressure, and reduced coronary perfusion (Sultan, Acharya, and Hynes 2020; Sultan, Concannon et al. 2020; Hynes et al. 2020; Sultan Barrett et al. 2020a, b, c). However, there is little insight in the surgical community about adverse cardiac remodelling after aortic stenting. Aortic interventionalists focus on the morphological adaptation of the endograft to the aortic wall and follow-up is purely focused on maintaining stent-graft position and avoiding expansion of the aortic sac. They continue to pursue these goals even if it means further interventions with additional endograft components, stenting or coiling, despite the resultant deleterious effect on cardiac, cerebral, renal and mesenteric haemodynamics. Medical device companies should contemplate that endografts significantly alter ventriculo-aortic interactions due to the mechanisms outlined in this paper. Contemporary endografts are not fit for purpose, especially in younger patients who have endografts implanted to treat thoracic aortic trauma near the heart (Sultan et al. 2019; Sultan Acharya and Hynes 2020; Hynes et al. 2020; Sultan, Concannon et al. 2020).

In this study, the effect of stent-graft deployment in a patient-specific aorta is investigated, where mechanical properties of the vessel are calibrated from (Concannon et al. 2020) and (Concannon and McGarry 2021). Our simulations demonstrate that the degree of oversizing should be tailored given the age of the patient and the deployment location relative to the heart, and we highlight the significant issues associated with insufficient oversizing and reaching the DEAL. In each case, stenting does not alter the local LCR of the aorta, provided the new diastolic equilibrium point is along the unloading plateau. The mechanism by which stenting alters the vessel compliance is uncovered and results from the outward radial force of the device stretching the collagen fibres beyond their transition point in the *in vivo* nonlinear pressure–area curve, such that the aortic wall loses its HCR and operates only in the LCR throughout the entire cardiac cycle postoperatively. The test-bed developed in this study can be used to design the next-generation devices and to guide clinical intervention so that post-operative complications including device migration and cardiac mortality can be reduced.

## References

[CR1] Aaron BB, Gosline JM (1981). Elastin as a random-network elastomer: a mechanical and optical analysis of single elastin fibers. Biopolymers.

[CR2] Abbaszadeh, Shahin, Mahdieh Eslami, and Marzieh Nikparvar. 2019. “Aortic Dissection in an 11-Year-Old Boy: Case Report Abbaszadeh et Al Case Report Aortic Dissection in an 11-Year-Old Boy: Case Report Aortic Dissection in an 11-Year-Old Boy: Case Report.” *Iranian Heart Journal*. Vol. 20

[CR3] Abbott WM, Megerman J, Hasson JE, Italien GL, Warnock DF (1987). Effect of compliance mismatch on vascular graft patency. J Vasc Surg.

[CR4] Atti V, Nalluri N, Kumar V, Tabet R, Yandrapalli S, Edla S, Tripathi A (2018). Frequency of 30-Day readmission and its causes after endovascular aneurysm intervention of abdominal aortic aneurysm (from the nationwide readmission database). Am J Cardiol.

[CR5] Auricchio F, Taylor RL (1997). Shape-Memory alloys: modelling and numerical simulations of the finite-strain superelastic behavior. Comput Methods Appl Mech Eng.

[CR24] Aridi D, Hanaa N, Locham S, Nejim B, Ghajar NS, Alshaikh H, Malas MB (2018). Indications, risk factors, and outcomes of 30-day readmission after infrarenal abdominal aneurysm repair. J Vasc Surg.

[CR6] Back M, Kopchok G, Mueller M, Cavaye D, Donayre C, White RA (1994). Changes in arterial wall compliance after endovascular stenting. J Vasc Surg.

[CR7] Baila, S, A Parnia, C Panaite, and M Salagean. 2016. “Arterial Bypass – a Surgical Method in Treatment of Peripheral Arterial Obstructive Disease of the Lower Limbs |.” Romanian Journal of Cardiology. 2016. https://www.romanianjournalcardiology.ro/arhiva/arterial-bypass-a-surgical-method-in-treatment-of-peripheral-arterial-obstructive-disease-of-the-lower-limbs/.

[CR8] Barakat HM, Shahin Y, McCollum PT, Chetter IC (2015). Prediction of organ-specific complications following abdominal aortic aneurysm repair using cardiopulmonary exercise testing. Anaesthesia.

[CR9] Beach JM, Kuramochi Y, Brier C, Roselli EE, Eagleton MJ (2017). Durable outcomes of thoracic endovascular aortic repair with zenith TX1 and TX2 devices. J Vasc Surg.

[CR11] Belz GG (1995). Elastic properties and windkessel function of the human aorta Cardiovascular Drugs and Therapy.

[CR12] Ben-Shlomo Y, Spears M, Boustred C, May M, Anderson SG, Benjamin EJ, Boutouyrie P (2014). Aortic pulse wave velocity improves cardiovascular event prediction. J Am Coll Cardiol.

[CR13] Benetos A, Safar M, Rudnichi A, Smulyan H, Richard JL, Ducimetieère P, Guize L (1997). Pulse pressure: a predictor of long-term cardiovascular mortality in a french male population. Hypertension.

[CR14] Bischoff MS, Ante M, Meisenbacher K, Böckler D (2016). Outcome of thoracic endovascular aortic repair in patients with thoracic and thoracoabdominal aortic aneurysms. J Vasc Surg.

[CR15] Blankensteijn JD, Lindenburg FP, Van der Graaf Y, Eikelboom BC (1998). Influence of study design on reported mortality and morbidity rates after abdominal aortic aneurysm repair. Br J Surg.

[CR16] Bock S, De F, Iannaccone MD, Beule DV, Loo F, Vermassen BV, Segers P (2013). Filling the void: a coalescent numerical and experimental technique to determine aortic stent graft mechanics. J Biomech.

[CR17] Bustos CA, García-Herrera CM, Celentano DJ (2016). Modelling and simulation of the mechanical response of a dacron graft in the pressurization test and an end-to-end anastomosis. J Mech Behav Biomed Mater.

[CR18] Cheng D, Martin J, Shennib H, Dunning J, Muneretto C, Schueler S, Von Segesser L, Sergeant P, Turina M (2010). Endovascular aortic repair versus open surgical repair for descending thoracic aortic disease a systematic review and meta-analysis of comparative studies. J Am Coll Cardiol.

[CR19] Chuter T (2002). Stent-Graft design: The good, the bad and the ugly. Cardiovasc Surg.

[CR20] Concannon J, Dockery P, Black A, Sultan S, Hynes N, McHugh PE, Moerman KM, McGarry JP (2019). Quantification of the regional bioarchitecture in the human aorta. J Anat.

[CR21] Concannon J, Hynes N, McMullen M, Smyth E, Moerman KM, McHugh PE, Sultan S, Karmonik C, McGarry JP (2020). A Dual-VENC 4D flow MRI framework for analysis of subject-specific heterogeneous Non-linear vessel deformation. J Biomech Eng.

[CR22] Concannon J, McGarry JP (2021). Development of an FEA framework for analysis of subject-specific aortic compliance based on 4D flow MRI. Acta Biomaterialia.

[CR23] Conrad MF, Tuchek J, Freezor R, Bavaria J, White R, Fairman R (2017). Results of the VALOR II trial of the medtronic valiant thoracic stent graft. J Vasc Surg.

[CR10] de Hector BWL, Conti M, Kamman AV, Nauta FJH, Lanzarone E, Moll FL, van Herwaarden JA, Auricchio F, Trimarchi S (2017). Stent-Graft deployment increases aortic stiffness in an ex&nbsp;vivo porcine model. Annals Vasc Surg.

[CR25] Donas K, Schonefeld T, Schwindt A, Troisi N, Torsello G (2011). Successful percutaneous endovascular treatment of symptomatic infrarenal aortic stenosis caused by soft-plaque with the endurant stent-graft - pubmed. J Cardiovasc Surg.

[CR26] Donas KP, Usai MV, Taneva GT, Criado FJ, Torsello GB, Kubilis P, Scali S, Veith FJ (2019). Impact of aortic stent-graft oversizing on outcomes of the chimney endovascular technique based on a new analysis of the PERICLES registry. Vascular.

[CR27] Ferrari G, Balasubramanian P, Tubaldi E, Giovanniello F, Amabili M (2019). Experiments on dynamic behaviour of a dacron aortic graft in a mock circulatory loop. J Biomech.

[CR28] Ferruzzi J, Bersi MR, Humphrey JD (2013). Biomechanical phenotyping of central arteries in health and disease: advantages of and methods for murine models. Ann Biomed Eng.

[CR29] Figueroa, C.A., and Christopher K. Zarins. 2011. “Computational Analysis of Displacement Forces Acting on Endografts Used to Treat Aortic Aneurysms.” In *Biomechanics and Mechanobiology of Aneurysms. Studies in Mechanobiology, Tissue Engineering and Biomaterials*, 221–46.

[CR30] Geest JPV, Sacks MS, Vorp DA (2004). Age dependency of the biaxial biomechanical behavior of human abdominal aorta. J Biomech Eng.

[CR31] Greenberg RK, Qingsheng Lu, Roselli EE, Svensson LG, Moon MC, Hernandez AV, Dowdall J (2008). Contemporary analysis of descending thoracic and thoracoabdominal aneurysm repair: a comparison of endovascular and open techniques. Circulation.

[CR32] Guan Ying, Wang Lu, Lin Jing, King MW (2016). Compliance study of endovascular stent grafts incorporated with polyester and polyurethane graft materials in both stented and unstented zones. Materials.

[CR33] Hinchliffe RJ, Bruijstens L, MacSweeney STR, Braithwaite BD (2006). A randomised trial of endovascular and open surgery for ruptured abdominal aortic aneurysm - results of a pilot study and lessons learned for future studies. Euro J Vasc Endovasc Surg : The Off J Euro Soc Vasc Surg.

[CR34] Hosoda Y, Kawano K, Yamasawa F, Ishii T, Shibata T, Inayama S (1984). Age-Dependent changes of collagen and elastin content in human aorta and pulmonary artery. Angiology.

[CR35] Hountis P, Dedeilias P, Bolos K (2009). Acute aortic dissection in a young patient without marfan fibrillinopathy: a case report. Cases J.

[CR36] Hynes N, Berguer R, Parodi JC, Acharya Y, Sultan S (2020). Management of complicated aortic dissection: natural history, translational research, simulation, bioconvergence, clinical evidence and literature review. Italian J Vasc Endovasc Surg.

[CR37] Kadoglou NPE, Moulakakis KG, Papadakis I, Ikonomidis I, Alepaki M, Lekakis J, Liapis CD (2012). Changes in aortic pulse wave velocity of patients undergoing endovascular repair of abdominal aortic aneurysms. J Endovasc Ther.

[CR38] Kadoglou NPE, Moulakakis KG, Papadakis I, Ikonomidis I, Alepaki M, Spathis A, Karakitsos P, Lekakis J, Liapis CD (2014). Differential effects of stent-graft fabrics on arterial stiffness in patients undergoing endovascular aneurysm repair. J Endovasc Therapy : An Off J Int Soc Endovasc Spec.

[CR39] Kent KC (2014). Clinical practice. abdominal aortic aneurysms. N Engl J Med.

[CR40] Kim J, Cocciolone AJ, Staiculescu MC, Mecham RP, Wagenseil JE (2019). Captopril treatment during development alleviates mechanically induced aortic remodeling in newborn elastin knockout mice. Biomech Model Mechanobiol.

[CR41] Kim SY, Hinkamp TJ, Jacobs WR, Lichtenberg RC, Posniak H, Pifarré R (1995). Effect of an inelastic aortic synthetic vascular graft on exercise hemodynamics. Ann Thorac Surg.

[CR42] Kotsis T, Spyropoulos BG, Asaloumidis N, Christoforou P, Katseni K, Papaconstantinou I (2019). Penetrating atherosclerotic ulcers of the abdominal aorta: a case report and review of the literature. Vasc Spec Int.

[CR43] Lonn L, Dias N, Veith Schroeder T, Resch T (2010). Is EVAR the treatment of choice for aortoenteric fistula? - pubmed. J Cardiovasc Surg.

[CR44] Marder, Victor J., William C. Aird, and Gilbert C. White. 2012. *Hemostasis and Thrombosis: Basic Principles and Clinical Practice*. https://books.google.ie/books?id=tG4-BdaCGAUC&pg=PT3180&lpg=PT3180&dq=EVAR+%2240+years+of+age%22&source=bl&ots=jEPpOTiFbb&sig=ACfU3U1MHV6Wym5Xss16gVsSbqGU_6eb4w&hl=en&sa=X&ved=2ahUKEwiK2bOe2rrnAhVHXhUIHa-ZALcQ6AEwBXoECAsQAQ#v=onepage&q=EVAR %2240 years of.

[CR45] Martín CE, Forteza A, Pérez E, López MJ, Centeno J, Blázquez JA, de Diego J, García D, Cortina JM (2008). Predictors of mortality and reoperation in acute type-a aortic dissection surgery: 18 years of experience. Revista Española De Cardiología.

[CR46] McGarry JP, Fu J, Yang MT, Chen CS, McMeeking RM, Evans AG, Deshpande VS (2009). Simulation of the contractile response of cells on an array of micro-posts. Philos Trans Royal Soc a: Math, Phys Eng Sci.

[CR47] Mehigan DG, Fitzpatrick B, Browne HI, Bouchier-Hayes DJ (1985). Is compliance mismatch the major cause of anastomotic arterial aneurysms? Analysis of 42 cases. J Cardiovasc Surg.

[CR48] Moerman KM (2018). GIBBON the geometry and image-based bioengineering add-on. J Open Sour Softw.

[CR49] Morita S, Asou T, Kuboyama I, Harasawa Y, Sunagawa K, Yasui H (2002). Inelastic vascular prosthesis for proximal aorta increases pulsatile arterial load and causes left ventricular hypertrophy in dogs. J Thorac Cardiovasc Surg.

[CR50] Morris L, Stefanov F, Hynes N, Diethrich EB, Sultan S (2016). An experimental evaluation of device/arterial wall compliance mismatch for four stent-graft devices and a multi-layer flow modulator device for the treatment of abdominal aortic aneurysms. Euro J Vasc Endovasc Surg : The Off J Euro Soc Vasc Surg.

[CR51] Morris L, Stefanov F, McGloughlin T (2013). Stent graft performance in the treatment of abdominal aortic aneurysms: the influence of compliance and geometry. J Biomech.

[CR52] Gundiah N, Ratcliffe MB, Pruitt LA (2009). The biomechanics of arterial elastin. J Mech Behav Biomed Mater.

[CR53] Nation DA, Wang GJ (2015). TEVAR: endovascular repair of the thoracic aorta. Semin Interv Radiol.

[CR54] Nauta FJH, de Beaufort HWL, Conti M, Marconi S, Kamman AV, Ferrara A, van Herwaarden JA, Moll FL, Auricchio F, Trimarchi S (2017). Impact of thoracic endovascular aortic repair on radial strain in an ***Ex vivo*** porcine model. Euro J Cardio-Thoracic Surg.

[CR55] Nolan DR, Ogden RW, Destrade M, McGarry JP (2014). A robust anisotropic hyperelastic formulation for the modelling of soft tissue. J Mech Behav Biomed Mater.

[CR58] Ramanan B, Fernandez CC, Sobel JD, Gasper WJ, Vartanian SM, Reilly LM, Chuter TAM, Hiramoto JS (2015). Midterm results of the use of low profile multibranched thoracoabdominal aortic stent grafts. J Vasc Surg.

[CR59] Salata K, Hussain MA, de Mestral C, Greco E, Aljabri BA, Mamdani M, Forbes TL, Bhatt DL, Verma S, Al-Omran M (2019). Comparison of outcomes in elective endovascular aortic repair vs open surgical repair of abdominal aortic aneurysms. JAMA Netw Open.

[CR60] Santos, I. C.T., Alexandra Rodrigues, Lígia Figueiredo, Luís A. Rocha, and João Manuel R.S. Tavares. 2012. “Mechanical Properties of Stent-Graft Materials.” *Proceedings of the Institution of Mechanical Engineers, Part L: Journal of Materials: Design and Applications*. 10.1177/1464420712451065

[CR61] Sarkar S, Salacinski HJ, Hamilton G, Seifalian AM (2006). The mechanical properties of infrainguinal vascular bypass grafts: their role in influencing patency. Eur J Vasc Endovasc Surg.

[CR62] Sher A, Tadros RO (2017). Endograft sizing for abdominal aortic aneurysms. Aortic Aneurysm InTech.

[CR63] Singh C, Wong CS, Wang X (2015). Medical textiles as vascular implants and their success to mimic natural arteries. J Funct Biomater.

[CR64] Sonesson B, Länne T, Vernersson E, Hansen F (1994). Sex difference in the mechanical properties of the abdominal aorta in human beings. J Vasc Surg.

[CR65] Sörelius, Karl. 2016. “Aortic Infections: The Nadir of Vascular Surgery.” http://urn.kb.se/resolve?urn=urn:nbn:se:uu:diva-300954.

[CR66] Spadaccio C, Nappi F, Al-Attar N, Sutherland FW, Acar C, Nenna A, Trombetta M, Chello M, Rainer A (2016). Old myths new concerns the long-term effects of ascending aorta replacement with dacron grafts not all that glitters is gold. J Cardiovasc Translational Res.

[CR67] Sugitani H, Hirano E, Knutsen RH, Shifren A, Wagenseil JE, Ciliberto C, Kozel BA (2012). Alternative splicing and tissue-specific elastin misassembly act as biological modifiers of human elastin gene frameshift mutations associated with dominant cutis laxa. J Biol Chem.

[CR68] Sherif S, Acharya Y, Hynes N (2020). The 4-D in management of complex aortic pathology. Italian J Vasc Endovasc Surg.

[CR69] Sultan S, Barrett N, Kamal MH, Tawfick W, Atteia EM, Clarkson K, Alawy M, Hynes N (2020). Staged hybrid single lumen reconstruction (tiger) in management of chronic symptomatic complex type b aortic dissection, techniques, and literature review. Ann Vasc Surg.

[CR70] Sherif S, Barrett N, Tawfick W, Parodi JC, Hynes N (2019). Contemporary abdominal aortic aneurysm devices three decades of research and development with big data why has the best graft not been produced yet a missed opportunity. Italian J Vasc Endovasc Surg.

[CR71] Sherif S, Concannon J, Mc G, Patrick J, McHugh PE, Barrett N, Hynes N (2020). Early results and lessons learned using the streamliner multilayer flow modulator in the management of complex thoracoabdominal aortic aneurysms and chronic symptomatic aortic Dissection. Italian J Vasc Endovasc Surg.

[CR72] Sherif S, Hynes N (2014). When not to implant the multilayer flow modulator: lessons learned from application outside the indications in patients with thoracoabdominal pathologies. J Endovasc Ther.

[CR73] Sultan, Sherif, Florian Stefanov, Liam Morris, and Niamh Hynes. 2015. “Abstract 19579: An Experimental Evaluation of the Compliance Mismatch Effects for Four Stent-Graft Devices and a Multi-Layer Flow Modulator (MFM) Device for the Treatment of Abdominal Aortic Aneurysms | Circulation.” 2015. https://www.ahajournals.org/doi/10.1161/circ.132.suppl_3.19579.

[CR74] Tiryakioglu SK, Tiryakioglu O, Turan T, Kumbay E (2009). Aortic dissection due to sildenafil abuse. Interact Cardiovasc Thorac Surg.

[CR75] Tremblay D, Zigras T, Cartier R, Leduc L, Butany J, Mongrain R, Leask RL (2009). A comparison of mechanical properties of materials used in aortic arch reconstruction. Ann Thorac Surg.

[CR76] Tshomba Y, Leopardi M, Ferrer C, Cao P, De Rango P, Verzini F, Melissano G, Coscarella C, Chiesa R (2017). Aneurysms of the thoraco-abdominal aorta: a comparison with propensity score between endovascular repair and open surgery. Ann Vasc Surg.

[CR56] Van Noort K, Holewijn S, Schuurmann RCL, Boersen JT, Overeem SP, Jebbink EG, Vermeulen JJM, Reijnen MMPJ, Slump CH, De Jean Paul PM, Vries.  (2018). Effect of abdominal aortic endoprostheses on arterial pulse wave velocity in an in vitro abdominal aortic flow model. Physiol Measurement.

[CR57] van Prehn J, Schlösser FJV, Muhs BE, Verhagen HJM, Moll FL, van Herwaarden JA (2009). Oversizing of aortic stent grafts for abdominal aneurysm repair: a systematic review of the benefits and risks. Euro J Vasc Endovasc Surg : The Off J Euro Soc Vasc Surg.

[CR77] Vernhet H, Demaria R, Juan JM, Oliva-Lauraire MC, Sénac JP, Dauzat M (2001). Changes in wall mechanics after endovascular stenting in the rabbit aorta: comparison of three stent designs. Am J Roentgenol.

[CR78] Yoneyama F, Sato F, Sakamoto H, Hiramatsu Y (2019). Preservation of the infected thoracic aortic endograft with thoracoscopic drainage and continuous irrigation. Gen Thorac Cardiovasc Surg.

[CR79] Zacharoulis AA, Arapi SM, Lazaros GA, Karavidas AI, Zacharoulis AA (2007). Changes in coronary flow reserve following stent implantation in the swine descending thoracic aorta. J Endovasc Ther.

[CR80] Zhou X, Yang F, Gong X, Zhao M, Zheng Y, Sun Z (2019). Development of new endovascular stent-graft system for type b thoracic aortic dissection with finite element analysis and experimental verification. J Mater Sci Technol.

[CR81] Zimmerman KP, Oderich G, Pochettino A, Hanson KT, Habermann EB, Bower TC, Gloviczki P, DeMartino RR (2016). Improving mortality trends for hospitalization of aortic dissection in the national inpatient sample. J Vasc Surg.

